# Refining understanding of working memory buffers through the construct of binding: Evidence from a single case informs theory and clinical practise

**DOI:** 10.1016/j.cortex.2018.08.011

**Published:** 2019-03

**Authors:** Pierre-Yves Jonin, Clara Calia, Sophie Muratot, Serge Belliard, Quentin Duché, Emmanuel J. Barbeau, Mario A. Parra

**Affiliations:** a Centre de Recherche Cerveau et Cognition, Université de Toulouse, CNRS CERCO, UMR 5549, Toulouse, France; b RISA, UMR CNRS 6074, VisAGeS U 1228, Inserm, INRIA, Université de Rennes 1, Rennes, France; c CHU Pontchaillou, Service de Neurologie, Rennes, France; d School of Health in Social Science, University of Edinburgh, Edinburgh, UK; e School of Social Sciences, Department of Psychology, Heriot-Watt University, Edinburgh, UK; f Universidad Autónoma del Caribe, Facultad de Psicología, Barranquilla, Colombia

**Keywords:** Working memory, Episodic buffer, Binding, Amnesia, Alzheimer's disease

## Abstract

Binding operations carried out in working memory enable the integration of information from different sources during online performance. While available evidence suggests that working memory may involve distinct binding functions, whether or not they all involve the episodic buffer as a cognitive substrate remains unclear. Similarly, knowledge about the neural underpinnings of working memory buffers is limited, more specifically regarding the involvement of medial temporal lobe structures. In the present study, we report on the case of patient KA, with developmental amnesia and selective damage to the whole hippocampal system. We found that KA was unable to hold shape-colours associations (relational binding) in working memory. In contrast, he could hold integrated coloured shapes (conjunctive binding) in two different tasks. Otherwise, and as expected, KA was impaired on three relational memory tasks thought to depend on the hippocampus that are widely used in the early detection of Alzheimer's disease. Our results emphasize a dissociation between two binding processes within working memory, suggesting that the visuo-spatial sketchpad could support conjunctive binding, and may rely upon a large cortical network including sub-hippocampal structures. By contrast, we found evidence for a selective impairment of relational binding in working memory when the hippocampal system is compromised, suggesting that the long-term memory deficit observed in amnesic patients may be related to impaired short-term relational binding at encoding. Finally, these findings may inform research on the early detection of Alzheimer's disease as the preservation of conjunctive binding in KA is in sharp contrast with the impaired performance demonstrated very early in this disease.

## 1 Introduction

Since its introduction in 1974, the Working Memory (WM) model proposed by Baddeley and Hitch (Baddeley & Hitch, 1974) has undergone revisions and refinements. A cognitive construct which has driven substantial amount of research and revisions of the model is binding, understood as the function that enables the integration of information from different sources during online performance (Zimmer, Mecklinger, & Lindenberger, 2006). To account for such an operation in WM, Baddeley proposed the episodic buffer, arguing that this may be the locus of binding functions (i.e., chunking) that were hard to accommodate in short-term memory (STM) buffers proposed earlier (Baddeley, 2000). This new component attracted considerable amount of attention leading to new questions about the structure and functions of WM and its neurobiological underpinnings (Baddeley, 2007a, Baddeley, 2007b, Baddeley et al., 2011). This paper reports on the study of a single case, patient KA, whose pattern of performance can shed new light on the ongoing debate about the function, structure, and neural substrate of WM as a workspace wherein different binding functions operate. Before we report on KA's history and assessment, we will briefly review the role that the construct of binding has played in shaping our understanding of WM. We will then address the literature reporting on neuroimaging and clinical studies that have sought evidence on the neural correlates of functions attributed to the episodic buffer and their vulnerability to brain damage and cognitive ageing. We will then introduce the current study emphasising the contribution that the evidence presented here can make to both understanding of the functional architecture of WM and refinement of memory assessment.

## 2 What has the construct of binding taught us about the functional organization of WM?

Baddeley (Baddeley, 2000, Baddeley, 2007a, Baddeley, 2007b) thought of the episodic buffer as a temporary store that integrates incoming information from other STM buffers and that retrieves them from long-term memory as unitary multimodal representations. Once formed, such bound representations become available to conscious awareness (Baddeley et al., 2011, Logie, 2011, Repovs and Baddeley, 2006). Repovs and Baddeley (2006) postulated that features held by the episodic buffer are stored in unitary representations either as integrated objects or as chunks (see Cowan, 2001, Cowan, 2010). Baddeley, Allen, et al. (2011) and Baddeley, Jarrold, et al. (2011)) argued that the episodic buffer has a central role in providing a multidimensional medium, allowing binding together chunks or features from different sources either visual or verbal, a process that requires executive control. In the verbal domain, Jefferies, Lambon Ralph, and Baddeley (2004) reported that under dual task conditions, recalling strings of unrelated sentences (i.e., scrambled words) was more disrupted than recalling random word lists, although on subsequent learning trials recall of the latter was also disrupted. These dual task effects were not observed for meaningful short stories. The authors concluded that whereas the requirement to integrate phonological with long-term linguistic information is not attentionally demanding, the integration of unrelated concepts is effortful. The processes supporting sentence recall reflect the contributions from both automatic linguistic functions and controlled binding functions operating on an attentionally limited WM component i.e., the episodic buffer. Vogel, Woodman, and Luck (2001) and Luck and Vogel (1997) investigated whether the integration of visual information in WM was cognitively demanding. Searching for evidence about the unit of representation of visual WM (i.e., integrated objects or individual features), the authors found that holding features integrated within unified object representations was not costlier than holding individuals features. They suggested that temporarily storing in visual WM objects defined by multiple features is a cost-free process, in as much as it does not consume additional WM capacity, and therefore the unit of representation of visual WM would likely be integrated objects. Wheeler and Treisman (2002) challenged this view by manipulating the change detection task in a way that required binding (i.e., swapping features between objects rather than adding new feature values as previously done by Vogel et al. (2001) and Luck and Vogel (1997). Under this task conditions binding in visual WM proved costly and such a cost varied depending on whether resources (i.e., feature dimensions) were drawn from the same or different pools (see Olson & Jiang, 2002 for further testing of this hypothesis). Allen, Baddeley, and Hitch (2006) decided to use the paradigm of dual-task interference to investigate whether binding in visual WM features drawn from different pools (i.e., colour and shape) was an automatic or a resource demanding function. Through a well-designed series of experiments (Allen et al., 2009, Allen et al., 2006, Karlsen et al., 2010), the authors demonstrated that this form of feature binding does not require executive resources above and beyond those needed to process single objects. This evidence was in line with the suggestions made by Vogel et al. (2001) and Luck and Vogel (1997) thus questioning the hypotheses that the episodic buffer is the seat of binding operations carried out in WM. Following this evidence, Baddeley, Allen, et al. (2011) and Baddeley, Jarrold, et al. (2011) revised the WM model to propose that this form of low-level feature binding may occur in other WM buffers such as the visuo-spatial sketchpad.

Allen et al. (2006) acknowledged that there are many different types of binding, depending on what stores, memory domains, or forms of representation are involved, and that visual feature binding is just one particular type. In fact, more recent studies have investigated whether other forms of binding which had been well characterised in long-term memory (e.g., associative learning or relational long-term memory binding; Mayes et al., 2007, Moses and Ryan, 2006) would operate in WM under the same cognitive constraints. This research has demonstrated that forms of memory binding well investigated in long-term memory seem to share functional properties when carried out in WM. Single case and neuroimaging studies have consistently demonstrated that two forms of memory binding, namely relational and conjunctive, known to dissociate in long-term memory (Mayes et al., 2007, Moses and Ryan, 2006), also dissociate in WM. When the features to be bound share internal relationships (features are part of the same object, e.g., coloured shape), conjunctive binding is involved, whereas relational binding refers to the processes linking features that share external relationships (i.e., features to be bound are part of distinct objects, e.g., face-name). These findings have shed new light on the neuroanatomical organization of brain networks subserving WM binding buffers. We review this evidence in the next section.

## 3 Neuroanatomy of WM buffers mapped through the construct of binding

The distinction between relational and conjunctive binding made in long-term memory (Moses and Ryan, 2006, Olsen et al., 2012) has also been investigated in WM. For example, Prabhakaran, Narayanan, Zhao, and Gabrieli (2000) investigated relational binding in WM by asking participants to remember arrays of letters presented in different locations or just letters and locations while being scanned using fMRI. The authors found that a network involving frontal, parietal, and temporal regions supported task performance. They reported a neuroanatomical dissociation for features and bindings whereby the right frontal region was preferentially involved in the maintenance of integrated representations in WM, and posterior brain regions were preferentially involved in the maintenance of individual features. Baddeley (2000) acknowledged that this network would well be the neural correlate of the episodic buffer. It is well known that in addition to frontal and parietal region, relational binding functions carried in WM also rely on medial temporal lobe (MTL) structures such as the hippocampus. For instance, Piekema, Rijpkema, Fernandez, and Kessels (2010) found that intrinsic intra-item binding (a form of conjunctive binding) did not yield activation of medial temporal lobe (MTL) structures whereas inter-item binding (a form of relational binding) did. Parra, Della Sala, Logie, and Morcom (2014) reported that holding conjunctions of features in WM did not recruit the hippocampus but regions forming a frontal-parietal-occipital-temporal network (i.e., left dorsal premotor cortex/middle frontal gyrus, left inferior parietal lobule, and left fusiform gyrus). Taken together these findings and the proposal by Baddeley, Allen, et al. (2011) and Baddeley, Jarrold, et al. (2011), one could argue that different binding functions carried out in WM may rely on different networks subserving different buffers. For instance, while a frontal-parietal-MTL network could be the neural correlate of the episodic buffer (Baddeley et al., 2011a, Prabhakaran et al., 2000), the parietal-occipital-temporal network could be the correlate of the visuo-spatial sketchpad (Parra et al., 2014, Shafritz et al., 2002, Todd and Marois, 2005, Xu and Chun, 2006).

Studies of single clinical cases have supported this view. Baddeley, Allen, and Vargha-Khadem (2010) investigated patient Jon who suffered from bilateral atrophy of the hippocampus from birth and have shown preserved conjunctive binding function in WM. Parra et al. (2015) reported on case AE who after a right hippocampal infarct, which caused amnesia, presented with a dramatic deficit to hold relations of features in WM but completely normal abilities to hold feature conjunctions.

While these studies might suggest that some forms of STM binding could rely on the hippocampus (see also Bird and Burgess, 2008, Ezzyat and Olson, 2008, Finke et al., 2008, Hannula et al., 2006, Hartley et al., 2007, Kan et al., 2007, Nichols et al., 2006, Olson et al., 2006; Piekema et al., 2007), it remains unclear why, in other MTL damaged patients, objects-locations or drawings-locations binding maintenance at short delays have consistently been reported as preserved (Jeneson et al., 2010, Jeneson and Squire, 2011, Jeneson et al., 2012, Shrager et al., 2008; see also Squire, 2017 for an example in the verbal domain). These authors suggest that as long as the task procedure does not exceed WM capacity, patients with amnesia do not present any WM binding deficit (Jeneson & Squire, 2011), a view more compatible with the typical contrast between impaired long-term but preserved STM performance in amnesia.

However, the hypothesis of a dissociation between the active maintenance of conjunctions versus relations of features in WM found support in research on cognitive ageing. For instance, older adults present with relational binding deficits in both long-term memory (Naveh-Benjamin et al., 2007, Naveh-Benjamin et al., 2004, Old and Naveh-Benjamin, 2008) and WM (Cowan et al., 2006, Mitchell et al., 2000, Peterson and Naveh-Benjamin, 2016). However, they seem to retain the ability to process conjunctions features until late in life (Brockmole et al., 2008, Hoefeijzers et al., 2017, Parra et al., 2009). This dissociation has been explained by the atrophy that the hippocampus undergoes with ageing (Mitchell et al., 2006, Mitchell et al., 2000). Monti et al. (2015) highlighted the important contribution that the hippocampus makes to relational memory processing across a broad range of tasks that span multiple domains. However, regions of the brain that appear to support conjunctive binding functions in LTM (i.e., entorhinal and perirhinal cortices; Mayes et al., 2007) remain unaffected by age (Insausti et al., 1998). From these perspectives, conjunctive binding functions may be more reliable to inform about abnormal ageing variants than relational binding functions.

Parra and collaborators have thoroughly investigated this hypothesis. They have found that indeed conjunctive binding functions separate normal ageing from mild stages of dementia due to Alzheimer's disease (AD) earlier and more accurately than relational binding functions (Koppara et al., 2015, Parra et al., 2010a). Hence, considering that the visuo-spatial sketchpad appears to host automatic, low-level WM binding functions which are subserved by age-resistant brain regions, while the episodic buffer hosts relational binding functions which require the contribution of the hippocampus, a structure known to shrink with age, assessment of functions supported by the former buffer may offer better opportunities to contribute to the early detection of AD than of those supported by the latter buffer. Despite this evidence, consensus papers continue to recommend relational binding tasks, particularly long-term associative memory tasks, as markers for the early detection of AD (e.g., Free and Cued Selective Reminding Tests; Costa et al., 2017; but see Della Sala, Kozlova, Stamate, & Parra, 2017). This position follows the long-standing view that relational forms of episodic memory (e.g., associative learning) which are supported by the hippocampus are the earliest memory functions affected by this form of dementia (but see Didic, Barbeau, Felician, Tramoni, Guedj & Poncet et al., 2011). The evidence supporting this notion is rather scattered in the literature and no one single study has brought together these methodologies to test patients with hippocampal damage. Such research is needed to demonstrate if those relational memory functions assessed by tests recommended by guidelines do indeed rely on the hippocampus while conjunctive binding as assessed by the STM binding test does not (Parra, Abrahams, Logie, & Della Sala, 2010).

## 4 The present study

The contribution that studies of patients with brain lesions has made to shaping our understanding of the functional architecture of WM has been widely acknowledged (Baddeley, 2007c). This paper addresses the question of whether neuropsychological evidence drawn from a single case could inform about the functional organization of WM buffers, which host different binding functions. The aim of the paper is twofold. A theoretical aim focuses on investigating the hypotheses that dissociations of memory binding functions can be observed in patients with hippocampal damage, and that such dissociations would allow further assessment of recent hypotheses regarding the anatomo-functional architecture of WM buffers (Baddeley et al., 2011). An applied aim focuses on further investigating the hypothesis that memory tests which assess conjunctive binding functions of WM buffers – which are preserved in healthy ageing and severely affected in the preclinical stages of AD – do not tax the function of the hippocampus, whereas those which assess relational or associative binding functions do. To test these hypotheses, we chose a battery of neuropsychological tests, which are being recommended by recent guidelines and consensus papers as useful markers for the early diagnosis of AD (Costa et al., 2017). As we noted above, such tests have never been used before together within a common assessment protocol. We predicted that the study of patient KA (see Jonin et al., 2018 for an in-depth case report) who we introduce next, would allow us to gain new insights about the dissociable nature of WM binding buffers, their neuroanatomical underpinnings, and the implications of such evidence for the assessment of age-related diseases such as AD. To investigate these hypotheses, we assessed KA with two sets of memory tasks. First, we selected a series of three memory tasks consistently reported as tapping hippocampal-dependent processes. These tests were also chosen because of their diagnostic value in identifying early AD. Second, we assessed KA's ability to perform two experimental STM binding tasks designed to further dissociate relational and conjunctive binding functions. In the next section, we describe the case of patient KA, together with a detailed examination of the radiological findings. We then provide the description of the tasks used together with the rationale for their selection and the overall experimental procedure.

## 5 Materials & methods

### 5.1 Case description

KA is a right-handed man who was 36 years-old at the time of assessment. This patient was first seen in the memory clinic of Rennes University Hospital in 2009, when he complained of memory deficits since he was a child which was corroborated by his family. His only and notable antecedent was severe neonatal hypoxia, and his neurological examination proved unremarkable. However, clinical observation revealed obvious limitations in moment-to-moment memory: KA often repeats himself without any awareness and cannot orient himself in an unfamiliar environment. Initial neuropsychological assessment confirmed very severe and selective memory impairment, without any other cognitive deficit (see Table 1, and see Jonin et al., 2018 for details). A 44 points discrepancy was found between Intelligence and Memory Quotients (IQ & MQ), KA scoring 97 and 53, respectively. Patient KA received different neuropsychological assessments between March 2009 and July 2015 without any notable change. A psychometric confirmation of his severe amnesia finally came from his performance on the Rivermead Behavioural Memory Test, patient KA scoring 5 (profile score), which is twice lower from previously well-known cases of early-onset amnesia (e.g., Rosenbaum, Carson, Abraham, Bowles, Kwan, Köhler et al., 2011).

**Table 1:** Patient KA's neuropsychological background. For the sake of clarity, raw scores were converted to percentile rank scores based on available normative data.

Cognitive domains/tests	Raw scores	Percentile Ranks
**French National Adult Reading Test**		
Raw score, max = 40	21	
Estimated Full Scale IQ (mean = 100, SD = 15)	100	50
Estimated Verbal IQ (mean = 100, SD = 15)	100	50
Estimated Performance IQ (mean = 100, SD = 15)	101	50
**Intelligence/Wechsler Adult Intelligence Scale, III**		
**List of subtests**		
Vocabulary	36	50
Information	22	75
Comprehension	20	37
Similarities	23	63
Digit Span	13	16
Letter Number Sequencing	8	9
Arithmetic	13	37
Picture Completion	22	63
Digit Symbol - Coding	62	16
Block design	29	6
Matrix reasoning	22	63
Symbol search	29	25
**Standard scores, mean** = **100, SD** = **15**		
Verbal Comprehension	105	63
Perceptual Organization	93	32
Working Memory	84	14
Processing Speed	84	14
**Memory/Wechsler Memory Scale III**		
**List of subtests – raw score**		
Digit span – forward	5	7
Digit span - backward	5	41
Spatial span – forward	5	9
Spatial span - backward	4	16
Information and orientation	14	>56
Logical Memory I	11	.1
Face Recognition	36	25
Verbal Paired Associates I	4	1
Family Pictures I	8	0,1
Words List, 1st recall	9	23
Words List, Total recall	23	2
Letter Number Sequencing	8	9
Spatial Memory	12	9
Mental Control	35	95
Digit Span	13	9
Logical Memory II	1	.1
Logical Memory II, retention (%)	12.5	.1
Face Recognition II	39	50
Verbal Paired Associates II	0	.1
Family Pictures II	9	2
Words List II	0	.1
**Standard scores, mean** = **100, SD** = **15**		
Verbal Immediate Recall	58	.3
Verbal Delayed Recall	54	.1
Visual Immediate Recall	67	1
Visual Delayed Recall	75	5
Delayed Recognition	56	.2
Working Memory	77	6
**Attention & Executive Functions**		
**2 & 7 Ruff Selective Attention Test**		
Speed	267	45
Efficiency	1.166	57
**Verbal fluency**		
Letter P	20	46
Letter R	20	59
Fruits category	16	35
**Ruff Figural Fluency Test**		
Unique designs (raw score corrected for age & education)	74	21
Perseverative errors ratio (raw score corrected for age & education)	.086	68
**Trail Making Test**		
Part A (seconds)	33	72
Part B (seconds)	72	80
**Hayling Test**		
Part A, total response time (seconds)	8427	31
Part B, total response time (seconds)	8130	–
Part B, raw score	0	80
**Dual task interference paradigm**		
Mu index	92.02	50

#### 5.1.1 Radiological findings

**Fig. 1:**
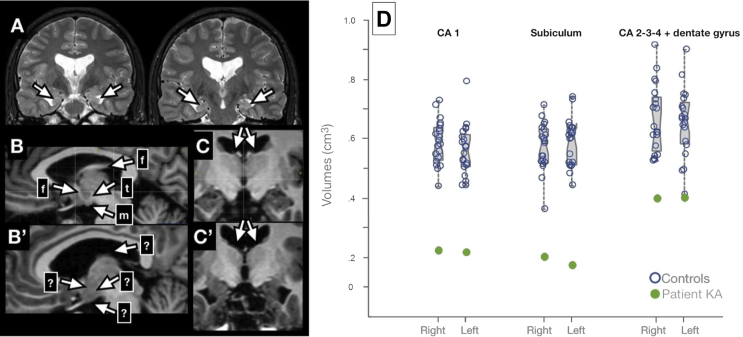
Structural MRI findings in the patient KA. (A) bi-hippocampal atrophy; (B) normal brain; (B’) atrophy of the fornix, mammillary bodies and mammillo-thalamic tract in KA's brain; (C) normal brain; (C’) atrophy of the anterior thalamic nuclei in KA's brain; (D) notched boxplots displaying normalized hippocampal volumes subfields in KA versus 20 matched control subjects. Notches represent 95% CI for the median; all comparisons being significant (Crawford's modified *t*-tests, all *p*-values <.05).

Visual examination of MRI scan (see Fig. 1) revealed bilateral atrophy of the hippocampal formation, together with severe atrophy of the fornix and bilateral anterior thalamic nuclei. The mammillary bodies as well as the mammillo-thalamic tract remained unidentifiable, an extremely rare condition across the literature.

To further examine patient KA's hippocampus, a dedicated high-resolution (.375*0.375*2 mm) proton-density-weighted MRI sequence was acquired on a 3T-scanner perpendicular to the long axis of the hippocampus. That sequence allowed to segment hippocampal subfields (CA1, Subiculum, and “other subfields”, i.e., CA2-3-dentate gyrus) according to a published protocol (La Joie, Fouquet, Mézenge, Landeau, Villain, Mevel et al., 2010) that was developed based on anatomical atlases (Duvernoy, 2005, Harding et al., 1998) and successfully applied to neurodegenerative disorders (La Joie, Perrotin, de La Sayette, Egret, Doeuvre, Belliard et al., 2013). Patient KA's volumes were compared to a group of 20 healthy males who were matched for age (mean: 28.4, SD: 3.4), but more educated than KA (years of education, mean: 14.5, SD: 3.0), after normalizing for total intracranial volume. This confirmed a severe bilateral hippocampal volume loss (volume loss exceeding 55%, z-score = –5.6), which was marked in every segmented subfield in both hemispheres, in particular in the CA1 and subiculum regions (see Fig. 1).

Altogether, clinical and neuroimaging data suggested that patient KA presented with a developmental amnesia syndrome (DA) as described in Vargha-Khadem et al. (1997) and Gadian, Aicardi, Watkins, Porter, Mishkin, & Vargha-Khadem et al. (2000). With patient HC (Hurley et al., 2011, Rosenbaum et al., 2011), KA's level of memory impairment is amongst the most severe ever reported across prior cases with DA. Moreover, and as recently reported (Dzieciol, Bachevalier, Saleem, Gadian, Saunders, Kling Chong et al., 2017), brain abnormalities in KA extended beyond the hippocampal formation, with the involvement of diencephalic structures and thalamus nuclei, suggesting that the whole hippocampal system has been compromised (for further details and a cortical thickness analysis, see Jonin et al., submitted).

### 5.2 Memory tasks

#### 5.2.1 Hippocampal-dependent tasks

We selected a set of three memory tasks based on their robust accuracy in identifying early AD: a Paired Associates Learning task (“PAL”) similar to that incorporated in the CANTAB (Sahakian et al., 1988), the Free and Cued Selective Reminding Test (FCSRT) (Buschke and Grober, 1986, Grober and Buschke, 1987) and the 4 mountains test (“4 MT”) (Hartley et al., 2007). We further describe each of these tasks and provide a brief overview of the evidence accumulated for (1) their relative specificity in identifying hippocampal damage and (2) their efficiency in identifying early AD.

*The PAL is* a visuo-spatial associative learning task requiring participants to encode series of object-location associations, then to recall the correct locations when cued with the object. We devised a PAL task similar to that reported by CANTAB, which was implemented with E-Prime 2.0 software (Psychology Software Tools, 2013) as follows. In the study phase, participants were asked to carefully look at an array of 8 boxes, one of which would contain an abstract object, visible during 2 sec. Subjects were instructed to memorize the location of the object. Immediately after the presentation of the object-location association, the test phase started. The same array of 8 empty boxes remained on the screen, with the object displayed at the centre. The subject had to use the mouse to click on the location (box) where the object was presented during the study phase. After a practise trial instructing the subjects that the number of object-location associations would increase along the test, the first study phase started. A total of six different levels were used, each corresponding to a different number of object-location associations, from 1 to 6. To progress to the next level, subjects had to succeed in the current level, so that participants were allowed to repeat study and test phase until they had learned the associations, up to a maximum of 10 attempts, after which the task stopped. The locations of the objects were randomly chosen for each trial, and objects were randomly selected among a set of 8 different abstract coloured objects.

The PAL task assesses relational binding functions by requiring participants to bind together each target object with its correct spatial location. Arguably, such relational binding functions mainly rely on long-term memory. Several lines of research demonstrated that PAL performance is tightly linked to hippocampal functions. An fMRI study revealed a load-dependent hippocampal activation such as activation increased when the number of to-be-learned patterns increased (de Rover, Pironti, McCabe, Acosta-Carbonero, Arana, Moreen-Zamir et al., 2011) Besides, animal lesion studies which inspired the task development (Parkinson, Murray, & Mishkin, 1988) confirmed the reliance of PAL performance upon hippocampal integrity (Kim, Heath, Kent, Bussey, & Saksida, 2015). Moreover, PAL has been successfully used in identification of AD, at various stages of the disease, including very early stages (e.g., Sahakian et al., 1988, Swainson et al., 2001; see also (Barnett, Blackwell, Sahakian, & Robbins, 2016) for a complete review about PAL findings). Finally, the number of errors across all attempts during PAL has recently been shown to correlate with the available biomarkers of AD in a sample of patients with MCI, i.e., CSF levels of tau, P-tau, Ab42, and hippocampal volumes (Nathan, Lim, Abbott, Galuzzi, Marizzoni, Babiloni et al., 2017), strongly arguing for the use of that task to identify hippocampal abnormalities in the context of early AD.

*The FCSRT* is a multiple trials verbal learning task, involving successive free and cued recall of 16 target words (Grober & Buschke, 1987). We used the French version of the task, developed by Van der Linden et al. (2004), together with corresponding available normative data. Under explicit learning instructions, four written words are displayed on a sheet of paper. The subject is asked to point to and read out each word in response to its semantic category label (e.g., for the word kipper: “Can you point to the fish and tell me what its name is?”). This first stage of the task allows the experimenter to check that the words have been semantically processed. Immediately after the words have been correctly identified, a cued recall task is administered. Based on a semantic association between the cue and the target word (e.g., for the word kipper: “What was the name of the fish?”), this task measures the effectiveness of associative encoding processes. Once immediate cued-recall has been performed for the 16 words, and after a 20-sec verbal interference task, participants perform free recall trials, followed by selective cued recall trials (cueing is only provided for items not recalled during the previous free recall trial). This procedure (free recall + cued recall) is repeated three times, with a 20-sec verbal interference task between each trial, to avoid subvocal rehearsal. This same procedure (delayed free + cued recall) is repeated after a 20-min interval. Finally, the selective reminding method is used, so that, in all the cued recall tasks except the third one, the correct answer is given only if the participant fails to recall the target word.

Since its initial development (Buschke, 1984, Buschke and Grober, 1986, Grober and Buschke, 1987, Grober et al., 2010), the FCSRT has been extensively studied in clinical settings. Prior research confirmed its accuracy in identifying mild dementia due to AD (Mura et al., 2017, Grober et al., 2010), but also showed that cued recall accurately predicts conversion to dementia due to AD in individuals with Mild Cognitive Impairment (Sarazin, Berr, De Rotrou, Fabrigoule, Pasquier, Legrain, et al., 2007). This led some authors to propose that the FCSRT may detect the “Amnestic syndrome of the medial temporal type” as a core sign of prodromal AD (Sarazin et al., 2007). The relationships between hippocampal integrity and index scores from the FCSRT have been highlighted in correlational imaging studies. For example, total recall score (i.e., free + cued recall) correlated with hippocampal volume in patients with AD at the dementia stage (Sarazin, Chauviré, Gerardin, Colliot, Kinkingnéhun, Cruz de Souza et al., 2010). Conversely, hippocampal volume has recently been found to accurately predict cueing efficiency scores in a sample of controls subjects and patients with mild cognitive impairment (Quenon, Dricot, Woodard, Hanseeuw, Gilis, Lhommel et al., 2016). Moreover, the authors reported that MCI patients with supra-threshold amyloid-β load in the brain had impaired cue efficiency measures as well as total recall scores relative to MCI patients whose amyloid-β load did not reach the threshold. Finally, the FCSRT has recently been associated with very high sensitivity and specificity for prodromal AD, including in clinical samples with mixed aetiologies (Teichmann et al., 2017, Wagner, 2012). Available evidence therefore suggests that the FCSRT is very sensitive to the hippocampal dysfunction observed in early AD.

*The* “*Four Mountains Test*” (4 MT) is an immediate forced-choice visual recognition task requiring participants to encode pictures of artificial scenes (Hartley, Bird, Chan, Cipolotti, Husain, Vargha-Khadem, et al., 2007). Briefly, participants are instructed that they will be presented with a picture representing a landscape with mountains, which they must look at carefully to further recognize it among 4 pictures. Subject's attention is drawn to both the mountains shapes and their spatial layout at encoding, because at test, the target picture is presented from a different viewpoint than during encoding. Subjects are therefore instructed that they should encode both the individual components of the landscape (i.e., mountains) but also their spatial layout. After a series of 6 practice trials, 15 items are presented as follows: the target picture is presented for 8 sec, then a blank slide is shown for 2 sec, then the 4 pictures of the test phase are displayed on the screen during 20 sec. A 2-sec inter-stimulus interval precedes the next trial. Subjects respond by simply pointing to the picture they thought was shown during the study phase. Because all landscapes pictures included 4 mountains, and because the viewpoint of the target picture differed between study and test, this task requires binding processes to allow the integration of mountains shapes with their relative placement. Such a topographical memory task therefore involves object-location associations learning (i.e., relational binding). Consistently with the “cognitive map” theory (O'Keefe & Nadel, 1978), these spatial relational processes were shown to heavily rely on hippocampal functioning. Lesion studies for example have highlighted the sensitivity of that task to amnesia following selective hippocampal damage (Hartley et al., 2007), and hippocampal volumes were found to correlate with task performance in healthy subjects as well (Hartley & Harlow, 2012). Accordingly, this task has been successfully used to identify early AD, among both healthy controls and other degenerative diseases (Bird et al., 2010, Chan et al., 2016, Pengas et al., 2010). Moreover, it has recently been found that the 4 MT score correlated with CSF tau-levels and further predicted conversion to dementia in a small sample of patients with Mild Cognitive Impairment (Wood, Moodley, Lever, Minati, & Chan, 2016).

#### 5.2.2 Conjunctive and relational memory binding tasks

**Fig. 2:**
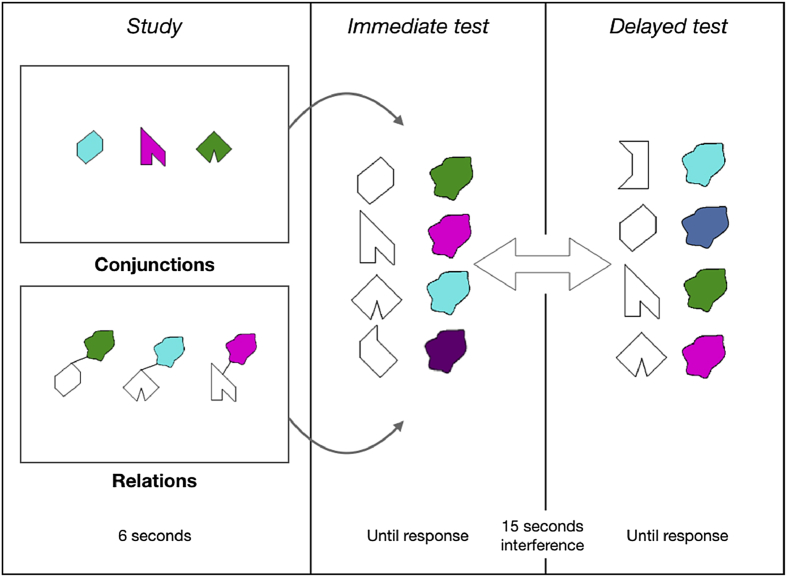
Illustration of the Visual Memory Binding Tasks (VMBT) for one trial. Subjects had up to 6 sec to encode either shape-colours relations or conjunctions, before being presented with an immediate forced-choice recognition test for individual features (shapes, colours), and the combinations. Study and immediate test phases were repeated until the participants reached 3 consecutive successful attempts. Then, after a 15 sec delay filled with verbal interference, the same test phase was repeated.

A set of eight polygons and a set of eight non-primary colours were used (see Parra et al., 2010a, Parra et al., 2010b) to create visual arrays presented during two Visual Memory Binding Tasks (VMBT), one tapping conjunctive and the other one relational binding functions. Fig. 2 illustrates the experimental procedure for one trial. A total of 6 trials were used for each condition (i.e., relational and conjunctive).

*In the conjunctive task*, 3 polygons randomly selected from the set of eight polygons (hereafter called “shapes”) were filled with 3 colours also randomly selected from the set of eight colours. These conjunctions were displayed for encoding for 6 sec on a white background. An empty white screen followed for 1 sec, preceding the test screen. At test, 4 shapes (3 targets + 1 distractor shape) were vertically presented on the left side of the screen, and 4 coloured blobs (3 targets + 1 distractor) were also vertically presented on the right side. Participants had to select using the mouse each target shape and its corresponding colour with reference to the study phase. After a minimum of three consecutive correct attempts (i.e., study + test phases with 100% accuracy), the task progressed to a delay period (15 sec), during which participants were asked to repeat out loud the letter “D” for 15 sec. If after 10 attempts participants failed to correctly learn the bindings between shapes and colours, the task progressed to the retention stage. After the filled retention interval, the test phase was administered once more (delayed component). Shapes and colours positions were randomly changed in the two recognition sets, so that position of test items could not be used as a memory cue. A series of 6 trials were administered, and instructions were summarized between trials for the amnesic patient. This resulted in a total of 18 shape-colour combinations to be learned. Shapes and colours were randomly chosen from the set of eight polygons and eight non-primary colours, thus resulting in different target and distractor sets for each participant.

*In the relational task*, the same procedures described above were used except that shapes and colours formed associations rather than conjunctions. A small black line joined the shape and its paired colour blob. The relational task was always administered before the conjunctive task for the entire sample.

For each memory binding task, several variables were considered regarding immediate (i.e., WM) and delayed (i.e., LTM) performance. Regarding WM, we first measured the recognition accuracy at the first immediate attempt, both for combinations (i.e., features conjunctions or relations) and individual features (i.e., shapes and colours). Maximum scores were 18 for each variable. We then computed the number of attempts required to reach the learning criterion, defined as the number of times the study + test phase had to be repeated to reach 100% accuracy (i.e., correctly selecting the 3 shapes and their corresponding colours). Finally, accuracy across all learning attempts was also computed, defined as the percentage of correct recognitions for either individual features (i.e., shapes and colours recognition accuracy) or combinations (recognition accuracy for conjunctions of features and for relations of features) across all trials. Regarding LTM, recognition accuracy scores for delayed task performance were also recorded, again for individual features and for combinations of features.

Our protocol therefore included of a set of three memory tasks that have proved sensitive to the early stages of AD as well as to be reliant on the hippocampal functioning. Of the three that assessed relational binding functions, two required to bind together an item with its location (i.e., PAL & 4 mountains test) and one required to associate words and semantic categories (i.e., semantic associative memory). Due to the selective damage to the hippocampal system sustained by patient KA, he should not succeed in any of these tasks. Regarding the contrast between conjunctive and relational memory binding procedures, patient KA's performance should be equally impaired after a delay in both conditions, because they will both rely on LTM processes to support retention of feature bindings. However, as stated in the Introduction, if hippocampal processing does not support conjunctive but only relational WM binding, KA performance on the first attempt of the relational task should be impaired but it should be normal on that of the conjunctive task.

### 5.3 Participants

A group of 15 male healthy participants (mean age = 36.1, SD = 3.31, range = 32–42; mean years of education = 12.5, SD = 2.33, range = 9–16) signed an informed consent to participate in the present study. Such a sample size and matching parameters are in line with prior cases studies on that topic, which generally involved a smaller control group (Allen et al., 2014, Baddeley et al., 2010, Ezzyat and Olson, 2008, Jeneson et al., 2010, Jeneson and Squire, 2011, Jeneson et al., 2012, Parra et al., 2009, Parra et al., 2015, Shrager et al., 2008). They were matched to patient KA for age and education (bilateral *p* values > .1). All participants signed an informed consent for the study, which was performed in accordance with the 1964 Declaration of Helsinki principles.

### 5.4 Procedure

All participants were assessed in a quiet room, free of any interference. The order of the tasks was fixed as follows: 1) PAL task; 2) VMBT-relational; 3) VMBT-conjunctive; 4) Four mountains test. The FCSRT was administered to patient KA as part of another testing session, and his scores were compared to available normative data, as stated above.

### 5.5 Statistics

Patient KA's performance was compared to that of healthy controls by means of Bayesian single-case statistical methods taken from (Crawford & Garthwaite, 2007). This approach allows controlling for type I errors when comparing a single case to a typically small sample of matched controls subjects. It further provides a Bayesian *p* value together with a Point Estimate (PE) of the abnormality of a given score, associated with the 95% credible interval for the estimation. The Bayesian PE directly provides an estimation of the percentage of the control population susceptible to obtain a lower (or, in case of measures like RTs or total number of attempts to reach learning criterion, a higher) score, than the case's score. Besides, Bayesian Standardized Difference Tests (BSDT) were applied to compute the probability that differences observed in the patient KA between conjunctive and relational binding performances could be observed in the reference population. Again here, a Bayesian *p* value is provided together with a PE corresponding to the percentage of the reference population susceptible to obtain a larger discrepancy, associated with a 95% confidence interval for the PE. Unless otherwise specified, one-tailed tests were used given the expectation of patient KA being impaired. Note that for the Free and Cued Selective Reminding Test, we made use of available normative data. Non-parametric testing was used to examine whether control participants performance differed between the two binding tasks (Wilcoxon signed-rank test), or whether some correlation between relational and conjunctive binding tasks could be found (Spearman's rank correlation coefficient). Finally, for the sake of clarity we have run a Monte-Carlo simulation (N = 10,000) that allowed us plotting the chance levels for each relevant scores of the two experimental tasks (see Fig. 4), thus making it easier for the reader to interpret the controls' and the patient’ scores.

**Fig. 3:**
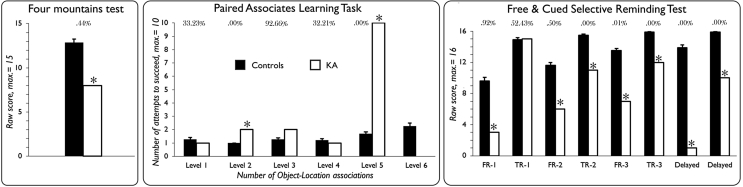
Patient KA's performance for the hippocampus-dependent memory tasks. Asterisks mark impaired scores (one-tailed Bayesian tests). Percentages correspond to the Bayesian Points Estimates of the proportion of the normal population susceptible to obtain either lower (Four mountains test & FCSRT) or higher (PAL task, total number of learning attempts required to succeed) scores. See text for detailed results. (FR-1 = Free Recall, first attempt; TR-1 = Total Recall (i.e., free + cued), first attempt).

**Fig. 4:**
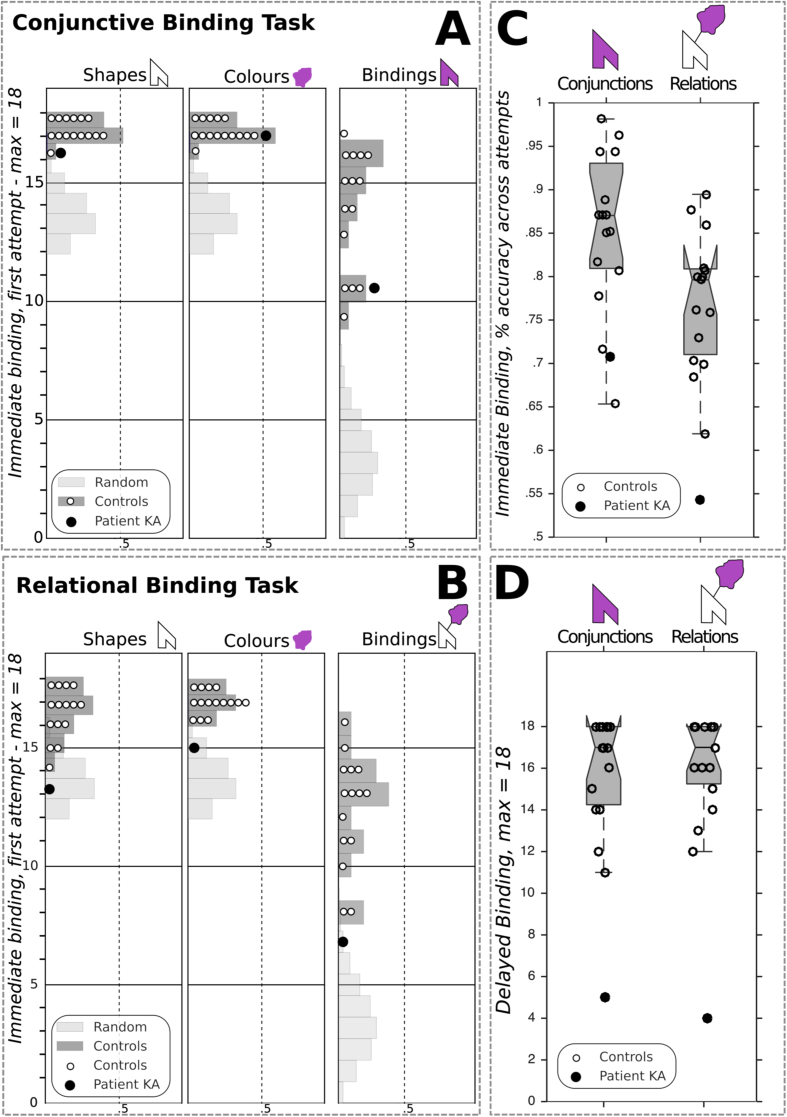
Visual memory binding performance of patient KA. Individual features and binding immediate recognition scores at first attempts for (A) the conjunctive and (B) the relational tasks. Random distribution in light grey corresponds to a Monte Carlo simulation ran with 10,000 iterations; Controls's scores distribution is displayed in dark grey; individual observations are plotted. (C&D) Notched boxplots showing the immediate binding accuracy (% correct) across all immediate attempts (C) and the delayed binding raw scores (D); notches represent the 95% CI around the median.

## 6 Results

### 6.1 Hippocampus-dependent memory tasks

#### 6.1.1 Paired Associates Learning task

Every control participant reached the last level of the task (i.e., 6 object-location associations to be remembered), meaning that they also successfully completed all the previous levels. Besides, each control subjects succeeded at the last level, with a maximum number of attempts of 4, and a maximum number of errors of 6. By contrast, patient KA succeeded the 4th level on the first attempt, but he failed to complete the 5th level, despite 10 consecutive attempts, making a total of 35 errors (see Fig. 4). Because this task requires participants to succeed at the current level before moving on to the next level (i.e., increasing the number of object-location associations), and because patient KA failed to complete the 5th level that was accurately completed by all controls, patient KA's performance can be considered as severely impaired. We also compared the number of learning attempts necessary to succeed in KA and controls (see Fig. 3). Patient KA did not need more attempts than controls to correctly recall 1, 3 and 4 object-locations associations, however he needed 2 attempts to succeed at level 2 (2 object-location associations), whereas all control subjects succeeded on their first attempt.

#### 6.1.2 Free and Cued Selective Reminding Test

Patient KA successfully performed the immediate cued recall phase, scoring 15/16, which did not differ from normative data [mean = 15.5, SD = .9; *p* = .*29*; PE = 29.45%, (17.48–43.41)]. Similarly, patient KA performed normally at the first total recall attempt (i.e., free + cued recall score) [controls' mean = 14.9, SD = 1.6; patient KA = 15; *p* = .*48*; PE = 52.43%, (32.82–71.52)]. However, we found KA to be impaired for all the other learning indexes. On average across the 3 immediate free recall trials, patient KA recalled 5.3 words [*p* = .*0025*; PE = .25%, (.00–1.34)]. The cueing procedure did not allow him to reach controls’ level, KA recalling 12.7 words on average across the three immediate total recall trials (i.e., free + cued recall score, max. = 48) [*p* = .*0049*; PE = .49%, (.02–2.25)]. Finally, delayed recall scores were also impaired in KA, either considering free [1/16, *p* < .*001*; PE = .00%, (.00–.00)] or total recall scores [10/16, *p* < .*001*; PE = .00%, (.00–.00)] (for detailed scores, see Fig. 3).

#### 6.1.3 Four mountains test

Patient KA scored 8/15 on the task, well below controls scores, with an estimate of less than .5% of the normal population expected to perform below KA's score [*p* = .*0044*; PE = .44%, (.00–3.04)] (see Fig. 3).

### 6.2 Visual memory binding tasks

Fig. 4 illustrates patient KA's performance for the immediate & delayed recognition parts of the visual binding tasks. Regarding *conjunctive binding*, patient KA performed at the bottom of the range of controls at first attempt and his score did not differ from controls [patient KA = 11/18; controls mean = 13.93, SD = 2.40; *p* = .128; PE = 13, (3.0–29.7)], and removing the lowest controls' score did not alter that result [n = 1, PE = 7.1%, (.9–20.7)]. While patient KA needed significantly more attempts than controls to succeed [patient KA = 24; controls mean = 18.93, SD = 1.87; *p* = .010; PE = 99%, (94.4–100.0) – to make it clear, such PE means that only 1% of the normal population is susceptible to need more attempts than KA], he proved as accurate as controls across all attempts [patient KA = .71%; controls mean = .85, SD = .09; *p* = .077; PE = 7.7%, (1.1–21.8)]. Given that control participants presented with an overall high level of performance in the conjunctive binding task, a ceiling effect may have reduced the probability of detecting impairment in the patient KA. To address this, we computed the skewness of the controls' scores distributions for both immediate binding score at first attempt and percent accuracy across immediate attempts. Skewness values are −.7638 (SE = .5801) and −.6959 (SE = .5801), respectively, which confirms that while the distributions are indeed negatively skewed, they do not show ceiling effects and remain in the acceptable range for normal univariate distributions (Gravetter & Wallnau, 2014). Finally, when specifically testing the corresponding distributions for normality (Shapiro–Wilk test), we found *p* values well above the alpha level (ps > .27). Overall, patient KA therefore seems to have preserved conjunctive binding scores, especially regarding the most relevant score, namely the accuracy at first attempt. Here, the patient performs in the low range of controls but remains unimpaired.

Turning to *relational binding*, patient KA was impaired at first relational binding attempt [patient KA = 7/18; controls mean = 12.33, SD = 2.35; *p* = .023; PE = 2.3%, (.1–9.9)]. He again needed significantly more attempts than controls to succeed [patient KA = 35; controls mean = 20.07, SD = 2.46; *p* < .001; PE = 100%, (99.2–100.0)], but remained well below controls' accuracy across all attempts [patient KA = .54%; controls mean = .77%, SD = .08; *p* = .007; PE = .8%, (.0–4.7)]. Interestingly, we found that patient KA's difference regarding the total number of immediate binding attempts between conditions fulfilled criteria for a strong dissociation, with a PE of 2.56% [.81–5.09]. In other words, these results suggest that the discrepancy observed in KA between conjunctive and relational learning attempts required for successful memory binding is likely to occur in less than 3% of the normal population. For that difference, the observed performance of KA was clearly out of the range of controls [KA = −11; controls' range = (–3; 4)]; and a similar difference was found for the total number of immediate trials [KA = −33; controls' range = (–9; 12)]. Importantly, controls performance did not differ between binding conditions, either for the first attempts scores (W = 55; *p* = .063) or the total number of attempts required (W = 12; *p* > .121), ruling out any significant difference in terms of task difficulty. Moreover, we failed to find any significant correlation between conjunctive and relational immediate binding scores (Spearman's *r* values ranging from .273 to .359, all *p* values >.05). To further emphasize our main finding, we estimated the uncertainty over the percentile rank of KA's scores at first attempts, for both conditions (Crawford, Garthwaite, & Slick, 2009). This led to a 95% Bayesian interval of 1.8–45.1 around the estimated percentile rank of 17 for the conjunctive condition, whereas the estimated percentile rank was 0 in the relational condition, with an associated interval of .0–15.2.

Considering delayed binding performance, patient KA proved severely impaired in both conditions to a similar extent (conjunctive: 5/18; relational: 4/18, *p* values <.001 in both cases, with PEs = .0%).

Patient KA's memory for individual features was also assessed, and compared with controls by use of two-tailed testing procedures for immediate scores, given the uncertainty about the status of recognition memory for single items in patients with amnesia (Holdstock et al., 2005, Yonelinas et al., 2010). Delayed scores were severely impaired in both conditions, with PEs ranging from 0 to 3.43%, all *p* values being well below 5%.

Considering immediate performance, patient KA presented normal scores at first recognition attempt for individual features in the *conjunctive* binding task (Shapes: patient KA = 16; controls' mean = 17.33, SD = .62; *p* = .057; Colours: patient KA = 17; controls' mean = 17.27, SD = .59; *p* = .665), but he was impaired in the *relational* task [Shapes: patient KA = 13; controls' mean = 16.6, SD = 1.24; *p* = .014; PE = .69%, (.00–4.32); Colours: patient KA = 15; controls' mean = 17.07, SD = .07; *p* = .013; PE = .63, (.00–3.99)]. Similarly, when considering overall accuracy across recognition trials for both shapes and colours as individual features, the *relational* binding task yielded impaired performance [Shapes: patient KA = .80; controls' mean = .95, SD = .04; *p* = .003; PE = .14%, (.00–1.14); Colours: patient KA = .90; controls mean = .96, SD = .02; *p* = .018; PE = .91, (.00–5.27)]. However in the *conjunctive* task, only Shapes recognition accuracy across trials proved impaired [patient KA = .90; controls mean = .98, SD = .03; *p* = .022; PE = 1.09%, (.01–5.98)], whereas Colours recognition remained in the fully normal range (patient KA = .97; controls mean = .97, SD = .02; *p* = .92). To sum up these findings about recognition accuracy for individual features, we found that in the conjunctive binding task, patient KA could normally hold individual colours and shapes in WM. By contrast, the patient failed to recognize individual features (either Shapes or Colours) in the relational binding task at first attempt, and he remained well below the controls’ level across further learning attempts.

Altogether, we therefore found patient KA to be severely impaired for both binding conditions after a delay filled with verbal interference. However, STM binding performance differed according to the task condition, since the patient completed the conjunctive task in the low but normal range, but was clearly impaired in the relational task. Moreover, the observed difference between the numbers of attempts required for learning shape-colours associations in the relational versus conjunctive binding conditions fulfilled the criteria for a strong dissociation. In fact, KA needed 75% more trials than controls to successfully learn the relational bindings while 26% more attempts were enough for the conjunctive bindings.

Nonetheless, the low-range score of the patient at first attempt in the conjunctive binding condition may suggest that, rather than being impaired in the relational WM binding task only, KA is also unable to normally hold conjunctions of features in WM. We therefore ran an additional conjunctive binding experiment, with a larger sample of controls subjects, to independently assess whether the patient's ability for visual WM conjunctive binding is truly preserved.

### 6.3 Additional visual memory binding task

Patient KA underwent a last visual WM binding task including three conditions: STM for individual features (Colours or Objects) and STM for conjunctive binding (i.e., Colours–Objects combinations). The task has been described in details elsewhere (Hoefeijzers et al., 2017). Briefly, the procedure was very similar to the binding tasks described above. In the critical binding condition, participants were presented with a pair of coloured objects (line drawings of living or manmade entities), with the same timing as in the other binding experiments (i.e., 1.5 sec per feature at study). An immediate recognition test involved the reconstruction of the object-colours bindings exactly as described in previous sections: participants had to select among a similar number of targets and lures, objects and their colours as they were presented during the study phase. For the individual features conditions (i.e., Objects or Colours), the study arrays included 4 items, while 2 items (i.e., two Object–Colours pairs) were used in the binding condition; the same number of lures was added at test. The number of features to be hold in STM was kept constant across conditions, and 8 trials were performed for a total of 24 STM test trials. For the purpose of the present study, we will focus on the immediate test scores, namely those corresponding to the first attempt score described in the previous section (6.2.).

The scores of patient KA were compared to those of 32 healthy controls matched for age and education (see Hoefeijzers et al., 2017), using the same statistical methods as the ones described in previous sections. One limitation that must be stressed here is that the control participants were Spanish-speaking Columbian subjects, while patient KA is a native French-speaker; note however that the set of objects was taken from the International Picture Naming Project (https://crl.ucsd.edu/experiments/ipnp/), thus limiting cultural biases. Patient KA performed very well for all conditions, with accuracy scores ranging from 93.75 to 100%, [Colours-only: patient KA = 93.75; controls' mean = 91.41, SD = 7.75; *p* = .768; PE = 61.6%, (47.8–74.4); Objects-only: patient KA = 100.00; controls' mean = 92.68, SD = 8.35; *p* = .394; PE = 80.3%, (67.8–90.0); Objects-Colours binding: patient KA = 100.00; controls’ mean = 93.164, SD = 9.31; *p* = .475; PE = 76.3%, (63.2–86.9)].

These results provide additional evidence suggesting that patient KA is not impaired in the STM conjunctive binding of pairs of individual features (Object–Colours in that case). Moreover, STM for individual features also was preserved, consistently with the results of the conjunctive memory binding task described in the section 5.2.2.

## 7 Discussion

The present single case study was set out to investigate two questions. At a theoretical level, we investigated whether binding functions carried out in WM could dissociate after hippocampal damage. We sought evidence that could inform recent hypotheses regarding the anatomo-functional architecture of WM buffers. At the applied level, we investigated whether the forms of binding which have proved sensitive and specific to AD are equally relying upon the hippocampal system.

We presented the case of patient KA with a syndrome of developmental amnesia due to bilateral hippocampal atrophy as well as severe atrophy of the anterior thalamus and diencephalic structures. Such a pattern of extensive and selective damage to the whole extended hippocampal system, leaving anterior subhippocampal structures intact, represents a unique opportunity to deepen our understanding of how that system contributes to binding functions. Based on current views and debates about (1) the role of the hippocampus and related MTL structures in WM binding functions, (2) tests devised to assess hippocampal functions in the early detection of AD, (3) and the vulnerability of these brain regions (MTL) to the neurodegenerative course of such a type or dementia, we thought KA's assessment would likely provide evidence to help address some of these outstanding issues. Three main findings resulted from KA's assessment. First, as predicted, patient KA proved severely impaired across three memory tasks selected for their known sensitivity to hippocampal damage, often performing below the 1st percentile relative to controls. Second, patient KA was unable to hold in WM (1-sec delay) three shape-colours associations (relational binding), whereas he could hold three integrated coloured shapes (conjunctive binding) remaining in the normal range of performance. Third, when tested after a 15-sec delay filled with a verbal task, performance dramatically dropped close-to-floor levels, both during conjunctive and relational binding conditions. In the discussion that follows, we map these findings to the outstanding issues abovementioned.

### 7.1 Binding in LTM and WM: what is unique and what is shared?

Evidence from the literature has consistently indicated that the relational or associative binding functions of the hippocampus support memory operations carried out both in WM and in LTM. Olsen et al. (2012) suggested that processing stimuli relations may follow a continuum from the very early stages of information processing (i.e., perception) to the stable representation in memory (i.e., LTM). Our data seem to support this view. We have reported that after early damage to the whole extended hippocampal system, patient KA is unable to perform three associative memory tasks. These tasks require the binding of features that share external relationships, which defines relational binding function (object – location (PAL), word – word (FCSRT), mountain – location (4 mountains test)). Similarly, patient KA was found unable to hold in mind for 1 sec three associations (or relations) between a shape and a colour blob. It is well acknowledged that relational representations are the core elements supporting declarative, long-term memories (Cohen & Eichenbaum, 1993). These findings suggest that STM tasks tapping relational binding function share a common reliance upon hippocampal function with tasks assessing associative LTM. KA's impairment in the delayed recall trials (i.e., free and cued recall) from the FCSRT strengthens this idea. The fact that patient KA was unable to recognize such relationships after 15 sec filled with verbal interference therefore opens the question of whether relational LTM deficits found in patients with amnesia result from poor consolidation of such memory traces or just impaired associative encoding. The fact that patient KA shows an impaired ability to hold three shape-colour relations after a 1-sec delay and then also failed to retrieve them after a longer filled interval is suggestive of the latter. Overall, our findings thus speak for a common binding function, namely, relational binding, as responsible for associative learning impairments across test delays (i.e., STM & LTM), and highly dependent upon the hippocampal system. That interpretation implies a role of the hippocampal system for STM, which remains a matter of debate as we discuss in the next section.

### 7.2 The case for hippocampal involvement in relational WM binding

Prior neuropsychological evidence suggested that amnesic patients with damage thought to be limited to the hippocampal formation are perfectly able to hold relations between features at short delays (see Introduction section). For example, Shrager et al. (2008) found that 5 patients with damage limited to the hippocampal formation successfully maintained for 1 sec up to 6 relations between drawings and locations. Likewise, Jeneson et al. (2010) found that, relative to 9 controls, 3 patients with damage limited to the hippocampal formation (also included in Shrager et al., 2008) displayed normal performance when asked to replace up to 4 objects onto their correct location after 1-sec delay. Such discrepancies across the current and these earlier studies may be accounted for by differences in the paradigms used.

Shrager et al. (2008) used a PAL task notably different from our PAL task. In the test phase, subjects were asked to make a Same/Different judgement on one single probe (i.e., either correct or recombined object – location association). By contrast, the PAL task used here requires participants to recall the location of each target object (i.e., location recall). Thus, the procedure used by Shrager et al. (2008) does not allow definitive conclusion about how many single associations have truly been hold in WM, and may therefore have overestimated the actual WM binding performance. Moreover, Shrager et al. (2008) used a 3 × 3 grid whereas our PAL task involves a round-shaped array of eight boxes; finally, while concrete objects where used by Shrager et al. (2008), we used abstract designs. These latter features could be of great importance because the associations between concrete objects and easily nameable locations (e.g., “there is a car in the bottom right case”, or “the car is in 3, 3”) may have supported a unitization strategy at encoding, which has been shown to boost recognition, even in amnesia (e.g., Borders et al., 2017, Parks and Yonelinas, 2015, Quamme et al., 2007, Ryan et al., 2013). These differences may help explaining why by using an apparently similar PAL task to that reported here, Shrager et al. (2008) found their amnesic patients to perform far better (they all reached 6 object-locations associations) than patients with early Alzheimer's Disease (AD) in the PAL task, where these patients typically cannot reach the level 6 (e.g., Swainson et al., 2001). Accordingly, the fact that patient KA succeeded only up to 4 associations makes sense given that his amnesia if far more severe than people with early AD. Moreover, this apparent limit of 4 object-location associations in the case of KA fits well with the findings of Jeneson et al. (2010) in 3 patients with hippocampal damage, who also seemed to systematically fail beyond this number of associations. Nonetheless, these authors report on the preserved STM of these patients for three object-location relations. What, then, could account for KA's failure to hold only 3 relations of features within WM in our relational binding task?

One possibility is that in the Jeneson et al. (2010) experiment, participants could again rely upon unitization strategies at encoding, because 1) real, nameable objects were used and 2) rather than a Same/Different judgement at test, subjects had to replace the correct objects in their correct area on a table, defining errors as the deviations from exact locations measured in controls. In our relational binding task however, such unitization is far less likely because only abstract shapes and hardly nameable colours were used, and exact relations between shapes and colours was required at test. Furthermore, Jeneson et al. (2010) asked participants to encode a set of real objects displayed on a table, and immediate test was performed on another table where subjects were instructed to physically replace the objects by reference to the study phase. This, again, may have overestimated their performance because in such a task not only visual but also kinaesthesic and, as stated above, verbal codes may have been involved. Contrary to such procedures, our binding task only probed visual WM, with no spatial, verbal, or kinaesthesic components. Patient KA's impairment in relational WM for three relations also fits with the findings from Olson et al. (2006), who reported impaired ability to hold 3 object-location associations after a 1-sec delay in 4 patients with amnesia and damage thought to be limited to the hippocampus. Because the design was quite similar to the one used in Shrager et al. (2008), the source of such divergent findings remains unclear. Shrager et al. (2008) pointed out that the patients from Olson et al. (2006) lacked MRI quantitative arguments for the absence of extra-hippocampal damage, and suggested that the absence of self-paced pause between trials could have been confusing for amnesic patients (i.e., possible forgetfulness of the instructions). In the present case study, instructions were repeated to patient KA between trials, and whole-brain volumetry failed to find any abnormalities beyond the extended hippocampal system (Jonin et al., 2018).

An alternative interpretation for the WM binding deficits of amnesic patients that has consistently been proposed by some authors is that their failure is due to an impaired contribution of LTM. If the WM binding task requirements exceed STM capacity (see Jeneson & Squire, 2011), that contribution would be necessary to perform the task at normal levels. Obviously, any WM task involving supra-span capacity at least partly relies on LTM. However, we think that this interpretation is very unlikely in the present report, for three reasons. First, estimates of spatial and verbal (digit) spans in the patient KA consistently reached 5 units of information (see Table 1), which, at first sight, seems to exceed the STM capacity required to hold in mind 3 shape-colours relationships for 1 sec. However, we do acknowledge that spatial and digit spans are insufficient proxies to estimate the visual span for abstract shapes and colours involved in our tasks. Future studies should design dedicated span tasks suitable to the working memory procedure used. Second, patient KA succeeded on the PAL task up to the level 4: he successfully recalled 4 different locations when probed with the corresponding objects. This, again, suggest that his STM capacity for single objects and, in that case, for object-location associations, is above the required size of 3 required in our WM binding tasks. Third, if patient KA's STM capacity was to be exceeded in the relational binding task, it should also have been the case in the conjunctive binding condition. However KA remained unimpaired albeit in the bottom range of controls.

With respect to the possible contribution of LTM, Olsen et al. (2012) acknowledged that the hippocampus supports relational binding and comparison with or without conscious awareness for the relational representations that are formed, retrieved and/or compared. They suggest that for these binding and comparison functions the reach of the hippocampus may expand beyond LTM memory and underlies task performance in multiple cognitive domains. Considering this assumption, we cannot completely rule out some support from LTM to our WM relational binding task. Should that be the case, both individual features and their associations may have become vulnerable in KA, whose hippocampi were severely damaged.

Finally, an unexpected finding concerned patient KA's scores for individual features. We found that while colours and shapes were individually correctly recognized in the conjunctive binding condition (at first attempts), he failed to accurately recognize these very same features in the relational condition. A straightforward account for this result could be related to the fact that we did not counterbalance the order of the conditions across subjects, always starting with the relational task. This should be taken into account, e.g., in a replication study. Notwithstanding, this result is not in line with the idea that STM for single items should be preserved in patients with amnesia. Note however that KA's performance for shapes was indeed low, but well beyond chance levels (i.e., 80% and 90% correct, in the relational and conjunctive tasks, respectively). One way to account for this finding is to consider that the encoding of black outlined polygons may require relational processing. Analytical visual perceptual processing would be required to perceptually bind together the components of these meaningless shapes. Such additional perceptual processing at encoding may have interfered with the encoding of the individual features (i.e., the shape and the colour blob). Similarly, the need for binding features presented separately in space may on its own have interfered with the perceptual processing of the single features. However, when the features are presented bound together, or “unitized”, these interference effects are no longer expected. We can only speculate that this could account for a relative weaker performance of KA for individual features in the relational binding task. If correct, that interpretation would imply that the use of meaningful, rather than meaningless, shapes should have little impact on STM for individual features, independently of the binding condition. Support for this view comes from the findings of Baddeley et al. (2010) in another patient with developmental amnesia, patient Jon. The authors used meaningful shapes (diamond, cross, square, etc.) and found that Jon's STM for individual features was perfectly normal, in both relational and conjunctive binding conditions (see below for further discussion). Moreover, we think that our data are unlikely to be accounted for by some feature memory deficit in the patient KA because the shapes used in the Conjunctive and Relational binding tasks were the same. Had KA had a deficit in processing shapes in WM, this would have become apparent in both tasks not just in the relational task.

Finally, one has to consider that this task is not very suitable to assess memory for single features, which would ideally rely on recognition. By contrast, our procedure involves to retrieval of the binding/relation, thus requiring a reconstruction process. Recognition of individual features and reconstruction of features combinations are distinct processes. Previous studies relying on this task have only focused on the reconstruction element as this allows assessment of the core relational and conjunctive functions for which this paradigm was intended (Parra et al., 2015, Van Geldorp et al., 2015). This is the first study that reports on memory for single features during this reconstruction paradigm. We acknowledge that although KA's performance across a wide range of tasks seems to confirm the presence of relational memory deficits across memory domains (WM and LTM), future studies with more specific designs are needed to investigate whether and to which extent processing in WM relations but not conjunctions also renders memory for constituent parts more vulnerable in patients with hippocampal damage.

### 7.3 Conjunctive WM binding following hippocampal amnesia

While patient KA failed to hold relational information at both short and long delays, he performed within the controls’ range on the conjunctive binding task only when such bindings were held in memory for 1 sec. Several arguments reinforce our interpretation of impaired relational binding despite relatively preserved conjunctive binding in the patient KA.

First, the relational and conjunctive binding tasks we used are closely matched, but nonetheless failed to yield any significant statistical association in controls. This result adds to the past reports using similar procedure and generally speaking for the view of two distinct binding constructs (Parra et al., 2009, Parra et al., 2010a, Parra et al., 2010b, Parra et al., 2015).

Second, several arguments do not support the intuition that the relational task may be more complex than the conjunctive task. The task was designed to allow an encoding time of 1 sec per feature in the two conditions (see Fig. 2). We believe this is sufficient amount of time to successfully encode the to be remembered items regardless of perceptual differences across task conditions. The two tasks presented the same type and number of features; the need to associate or integrate them being the only difference between task conditions. We did not find evidence for a significant difference between the two tasks in controls. When computing the difference between raw binding scores at first trial in controls (Relational minus Conjunctive), we found a median score of 1, with 2 controls presenting a negative score, and a majority of controls presenting a score at or below 2. Finally, it is worth noting that the order of the tasks was kept constant across participants, who started with the supposedly more complex condition. These facts all converge to rule out a complexity account for our findings of impaired relational but preserved conjunctive WM binding in the patient KA.

Third, our testing procedure required participants to make a forced-choice recognition task for each individual feature as well as for the associations between these features. That is, even in the conjunctive condition, participants must have successfully encode both individual features and their associations to perform correctly, thus ruling out any strategy relying on single-feature encoding.

Fourth, study items were made of non-overlapped features (i.e., paired) in the relational binding condition and at test, recognition relied on two spatially separated sets of features which provided no cues to aid memory for relations or conjunctions (or even support from familiarity). Such design features make it very unlikely that a conjunctive strategy would aid performance on the relational condition of our paradigm. Moreover, the colours and the shapes were elaborated so that they both are very hardly nameable. Any encoding strategy based on encoding a single verbal token for a particular shape-colour association was therefore very unlikely. However, if some participants had to use such a strategy (resulting in some unitization of the features to be bound), one might expect this to facilitate the relational condition, but not the conjunctive condition where the features are presented already bound together. Altogether, we thus argue that our binding tasks do tap into non-overlapping working memory binding processes, which have been shown to dissociate in prior studies, and that the dissociation observed in KA is unlikely to be accounted for by different encoding strategies.

When shape-colour conjunctions were the memoranda, damage to the extended hippocampal system therefore left WM binding unimpaired. However, a longer retention interval of 15 sec filled with a simple verbal interfering task was sufficient to dramatically disrupt patient KA's ability to retain such conjunctions as indicated by performance far below controls' level. These observations are suggestive of a WM function independent from LTM, allowing only temporary storage of features that share internal relationships (i.e., conjunctive binding), and that do not rely on the hippocampal system function. Thus, while relational binding function seems to support the formation of both short- and long-term memories, low-level conjunctive binding seems to operate only within WM. Earlier neuropsychological and neuroimaging studies have provided support for such a view (for an overview, see Olsen et al., 2012), albeit they rarely directly investigated the contrast between relational and conjunctive binding functions. For example, patient AE (Parra et al., 2015) with unilateral right ischaemic lesions of the posterior thalamus, parahippocampal gyrus, and hippocampus presented with impaired WM relational binding leaving conjunctive binding unaffected. Importantly, this held even when using the same abstract shapes as we used in the present study, ruling out any subvocal rehearsal contribution to performance. It has been suggested that the neural underpinnings of LTM encoding may differ depending on the strategy used, either based on unitization or on relational binding, the former relying on perirhinal cortex activity (Davachi et al., 2003, Staresina and Davachi, 2006). Quite recently, event-related potentials at encoding brought evidence that these strategies might reflect two distinct and complementary learning systems, again relying upon distinct neural networks (Tu, Alty, & Diana, 2017). The discrepancies between our findings in patient KA and prior findings in patient Jon (see below) suggest that when the design of STM binding tasks makes it possible to use a unitization strategy, subhippocampal structures like the perirhinal cortex, preserved in both patients, could be sufficient to perform at a fair level. However, in that case, performance would reflect preserved unitization at encoding, rather than relational binding. An interesting possibility is that conjunctive binding and unitization share common properties, starting with a common neural substrate, but also a critical role in forming new representations for within-domain associations. Nevertheless, a question that remains is which cognitive system can support conjunctive binding and on which neural basis it relies, a question we address in the following section.

### 7.4 Which WM buffer supports conjunctive binding?

With respect to the cognitive substrates of conjunctive binding, Allen et al. (2006) demonstrated that feature binding in visual WM does not require executive resources above and beyond those needed to process single objects. This evidence already questioned whether the episodic buffer would be necessary for this form of binding. Besides, the hippocampus had been considered a binding device, which grants integrative abilities to the episodic buffer necessary for episodic memory formation (Prabhakaran et al., 2000, Jefferies et al., 2004, Baddeley et al., 2011). Therefore, being independent of both executive resources and the hippocampus leaves conjunctive binding functions carried out in WM in need of an alternative buffer. An obvious candidate is the visuo-spatial sketchpad, since it was assumed to support low-level binding functions as the ones needed to form object's identity (Staresina & Davachi, 2006). This would also fit with the last revision of Baddeley's model of WM (Baddeley et al., 2011), suggesting that low-level features binding do not rely upon the episodic buffer.

Turning to the potential neural underpinnings of conjunctive binding, an obvious candidate is the ventral visual stream, and particularly the perirhinal cortex, acknowledged as being the core substrate of the conjunctions of features that support objects’ recognition (e.g., Olsen et al., 2012, Staresina and Davachi, 2006), and being fully preserved in patient KA. The neuroimaging literature has also lent support to the idea that conjunctive binding within WM binding may be independent from the hippocampus. Parra et al. (2014) for example have shown that the active maintenance of conjunctions of features (shapes – colours) at short delays mainly relied upon a temporo-parietal network, associated with left frontal areas (precentral gyrus and premotor cortex), without involvement of the hippocampus. Similarly, Piekema et al. (2010) suggested that intra-item binding in WM (a kind of conjunctive binding) did not involve MTL activations. It could thus be speculated that different binding functions carried out in WM may rely on different networks subserving different buffers. While the episodic buffer would have a frontal-parietal-MTL network as a neural correlate (Baddeley, Jarrold, et al., 2011), the perirhinal cortex and a large frontal-temporal-parietal network could be the correlate of the visuo-spatial sketchpad (Parra et al., 2014, Shafritz et al., 2002, Todd and Marois, 2005, Xu and Chun, 2006), as the locus of conjunctive binding in WM.

### 7.5 Relational WM binding in developmental amnesia

We are aware of only one other study of WM binding in developmental amnesia. Baddeley et al. (2010) have extensively studied visual WM binding (i.e., shape-colour) in patient Jon, who suffers from developmental amnesia like patient KA, thus making their study very relevant to our own findings. Participants had to encode four shape-colours associations that where displayed simultaneously for 250 msec, but presented separated in space (i.e., a colour blob on top of an unfilled shape) to further make an Old/New judgement on a coloured shape (i.e., target or lure) used as the test probe at a 900 msec delay. Jon's performance averaged across 24 trials was in the fully normal range, and he even tended to outperform controls. It is therefore surprising that, with a very similar procedure, we found patient KA to fail the active maintenance of only 3 shape-colours associations. However, a single probe was always used with patient Jon, always presented as a conjunction (i.e., a coloured shape), which might have allowed the patient to rely upon a unitization strategy (see also Parra et al., 2015), which was not possible in our relational binding task where the reconstruction at test required KA to recognize the target single features and recall their bindings after 1-sec delay (see Brockmole and Logie (2013) and Hoefeijzers et al. (2017) for similar procedures). Moreover, Baddeley et al. (2010) used meaningful shapes (a cross, a diamond, a square, etc.). We therefore consider the possibility that other STM processes like subvocal rehearsal, depending on extra-hippocampal structures (Buchsbaum, Olsen, Koch, & Berman, 2005), might at least partly have contributed to Jon's performance, thus overcoming a relational binding deficit. Early support for this interpretation comes from studies showing that abstract shapes maintenance rapidly decays in amnesic patients (e.g., Butters, Lewis, Cermak, & Goodglass, 1973). By contrast, when testing patient KA with non-nameable polygons, such subvocal rehearsal is very unlikely, making our design possibly less contaminated by WM processes independent from binding itself.

### 7.6 Which memory binding function should we assess in AD and when?

The applied aim of the present study was to make the case of KA's assessment informative about the construct validity of tests devised for the early diagnosis of AD. We are not aware of prior studies systematically assessing the validity of hippocampus-dependent memory tasks used in the context of AD diagnosis by administering these tests to patients with amnesia (but see Hartley et al., 2007 for an exception with the 4 mountains test). We reasoned that if tests failed very early in the course of AD could be successfully performed after damage to the hippocampi, this would suggest a need to move from hippocampal-dependent memory tasks towards new tests, better suited to their early cognitive markers properties for AD.

The recommended tests for the assessment of early AD are tapping relational (or associative) binding processes. It is the case for tasks such as the PAL test from the Cambridge Neuropsychological Test Automated Battery (CANTAB; Sahakian, et al., 1988); the Face Name Associative Memory Exam (FNAME; Amariglio et al., 2012, Rentz et al., 2011, Free and Cued Selective Reminding test (FCSRT; Grober, Buschke, Crystal, Bang, & Dresner, 1988), Memory Capacity Task (MCT); Memory Impairment Screen (MIS; Buschke et al., 1999). People with AD typically show difficulty in these specific tasks. Such an associative memory deficit in AD is linked to the hippocampal stage which correspond to stage III or IV of Braak's scale (Braak, Thal, Ghebremedhin, & Del Tredici, 2011). This involves structures of the posterior MTL network (Didic et al., 2011), such as parahippocampal cortex, medial entorhinal cortex, posterior hippocampus and posterior cingulate cortex where neurofibrillary tangles develop later. These structures play a relevant role in context-rich memory tests (Didic, et al., 2011). The reason is that damage to the hippocampus and related structures at that stage of the disease prevents the formation and maintenance of new associations. These observations have been confirmed by a study conducted by Sperling, Bates, Chua, Cocchiarella, Rentz & Rosen et al. (2003), who observed a significant reduction of hippocampal activation during encoding of new face-names associations in patients with mild AD. Importantly, they observed that healthy elderly also presented significantly reduced hippocampal activity, albeit to a lesser extent than mild AD patients. These findings may explain the difficulties of the elderly in performing associative memory tasks, in accordance with the influential associative deficit hypothesis (Naveh-Benjamin, 2000). Thus, the tests involving relational binding function, including WM relational binding tasks, would present a sub-optimal specificity for the early diagnosis of AD.

In the present study, the fact that patient KA is impaired in the three tasks currently used in the early detection of AD brings evidence reinforcing the sensitivity of these tasks to the hippocampal stage of AD. Interestingly, the score of patient KA at the “4 mountains test” (8/15) exactly replicated an earlier finding with that task in patient Jon, and also fits with the cut-off score of 8 or below for differentiating between Mild Cognitive Impairment patients with or without positive CSF biomarkers for AD pathology (i.e., levels of β-amyloid_1-42_ and phosphorylated tau) (Chan et al., 2016). This confirms that WM relational binding function is very sensitive to hippocampal damage, either arising from early hypoxia as in patient KA, or from AD-related pathology at the hippocampal stage, or simply from ageing.

However, in the subhippocampal stages of AD, the early target of tau pathology is the entorhinal cortex (Van Hoesen et al., 1991, Juottonen et al., 1998). Several studies already demonstrated that the volume of the entorhinal cortex compared with the hippocampus volume is a more informative signal of conversion from MCI to AD (Dickerson et al., 2001, Shoghi-Jadid et al., 2002, deToledo-Morrell et al., 2004). It is also known that context-free tasks such as familiarity based-recognition memory (Barbeau et al., 2004, Besson et al., 2015, Haskins et al., 2008) could be suitable cognitive markers to probe the early dysfunction of brain areas that appear affected in this stage, but it is not clear how the different MTL areas are related to memory deficits in AD (Hoefeijzers, Calia, & Parra, 2016). Didic et al.’s model (2011) suggested an account for how memory systems are affected in the AD continuum. Early damage to the subhippocampal structures may determine impairments in context-free memory tests, while the hippocampus seems to be related to context-rich memory tasks. Interestingly, there is consistent evidence that WM conjunctive binding accurately detects AD in its earliest, preclinical, stages (Della Sala et al., 2017, Della Sala et al., 2012, Koppara et al., 2015, Parra et al., 2010a, Parra et al., 2010b, Parra et al., 2011, Rentz et al., 2013), while relational binding remains completely preserved. Furthermore, recent studies in cases of both familial and sporadic AD using electrophysiological techniques (EEG-ERP and brain connectivity), have reported both poor activation (Pietto, Parra, Trujillo, Flores, García, Bustin, et al., 2016) and connectivity (Parra, Mikulan, Trujillo, DellaSala, Lopera, Manes, et al., 2017) within the cortical network thought to underlie the visuo-spatial sketchpad. Considering that patient KA successfully performed the same WM conjunctive binding task as the one impaired in the preclinical stages of AD, while he was severely impaired on the WM relational binding task that is fully preserved at that stage of AD, we argue that conjunctive, not relational, binding function should be targeted for the early detection of AD. These observations in patient KA finally suggest that memory tests currently recommended for the diagnosis of AD (e.g., Costa et al., 2017) may actually lack specificity for the disease and, perhaps more importantly, miss their target as they may be sensitive to memory dysfunctions associated with late hippocampal stages, rather than early, subhippocampal stages.

## 8 Conclusion

We have reported on the case of patient KA, with a syndrome of developmental amnesia associated with selective damage to the whole extended hippocampal system. While the patient proved severely impaired in all tasks involving relational binding function, including WM tasks, he remained in the low to normal range in WM conjunctive binding tasks. Our findings therefore speak for a dissociation between STM binding functions after hippocampal damage, and inform the clinical assessment of early AD. Future studies will be needed to test the independence of conjunctive binding from the episodic buffer as well as its neural underpinnings, and to investigate whether, within WM, tasks tapping into the visuo-spatial sketchpad rather than the episodic buffer might offer better opportunities for the early detection of AD.
